# Inhalation of rod-like carbon nanotubes causes unconventional allergic airway inflammation

**DOI:** 10.1186/s12989-014-0048-2

**Published:** 2014-10-16

**Authors:** Elina M Rydman, Marit Ilves, Antti J Koivisto, Pia A S Kinaret, Vittorio Fortino, Terhi S Savinko, Maili T Lehto, Ville Pulkkinen, Minnamari Vippola, Kaarle J Hämeri, Sampsa Matikainen, Henrik Wolff, Kai M Savolainen, Dario Greco, Harri Alenius

**Affiliations:** Nanosafety Research Centre, Finnish Institute of Occupational Health, Helsinki, Finland; Pulmonary Division, Department of Medicine, University of Helsinki, Helsinki, Finland; Department of Materials Science, Tampere University of Technology, Tampere, Finland; Department of Physics, University of Helsinki, Helsinki, Finland

**Keywords:** Carbon nanotubes, Inhalation, Immune system, Transcriptomics, Inflammation, Allergic airway inflammation, Asthma

## Abstract

**Background:**

Carbon nanotubes (CNT) represent a great promise for technological and industrial development but serious concerns on their health effects have also emerged. Rod-shaped CNT are, in fact, able to induce asbestos-like pathogenicity in mice including granuloma formation in abdominal cavity and sub-pleural fibrosis. Exposure to CNT, especially in the occupational context, happens mainly by inhalation. However, little is known about the possible effects of CNT on pulmonary allergic diseases, such as asthma.

**Methods:**

We exposed mice by inhalation to two types of multi-walled CNT, rigid rod-like and flexible tangled CNT, for four hours a day once or on four consecutive days. Early events were monitored immediately and 24 hours after the single inhalation exposure and the four day exposure mimicked an occupational work week. Mast cell deficient mice were used to evaluate the role of mast cells in the occurring inflammation.

**Results:**

Here we show that even a short-term inhalation of the rod-like CNT induces novel innate immunity-mediated allergic-like airway inflammation in healthy mice. Marked eosinophilia was accompanied by mucus hypersecretion, AHR and the expression of Th2-type cytokines. Exploration of the early events by transcriptomics analysis reveals that a single 4-h exposure to rod-shaped CNT, but not to tangled CNT, causes a radical up-regulation of genes involved in innate immunity and cytokine/chemokine pathways. Mast cells were found to partially regulate the inflammation caused by rod-like CNT, but also alveaolar macrophages play an important role in the early stages.

**Conclusions:**

These observations emphasize the diverse abilities of CNT to impact the immune system, and they should be taken into account for hazard assessment.

**Electronic supplementary material:**

The online version of this article (doi:10.1186/s12989-014-0048-2) contains supplementary material, which is available to authorized users.

## Background

Allergic asthma is a chronic inflammatory disorder of the epithelial surfaces of the lung, characterized by airway hyperresponsiveness (AHR), mucus overproduction, pulmonary eosinophilia and allergen-driven T helper (Th) 2 lymphocyte polarization [1]. Coordinated production of the Th2 cytokines IL-4, IL-5 and IL-13, and production of allergen-specific IgE antibodies drives the allergic inflammation through the recruitment and activation of T-cells and eosinophils. Chronic Th2 inflammation may finally lead to structural changes in the airways in a process called airway remodeling [2]. Despite the involvement of several cell types, Th2 cells are considered to be pivotal in the pathobiology of asthma.

Asthma represents the most important chronic inflammatory disease of the lung and is the major childhood illnesses in Europe and in US. The incidence of asthma is steadily increasing in industrialized countries affecting about 5–10% of the population [3,4]. The reason of increased asthma prevalence is unknown but it has been linked with improved hygienic standards. This has been called the “hygiene hypothesis” arguing that early childhood exposure to microbes inhibits the tendency to develop allergic diseases [5]. On the other hand, a competing hypothesis suggests that more exposure to a wide variety of novel chemicals in the environment, in consumer products and in occupational settings, is the major cause for the sudden peak in asthma prevalence [6,7].

The unique properties of carbon nanomaterials and their applications have the potential for a remarkable technological and economic growth [8]. The ability to custom synthesize carbon nanotubes (CNT) with attached functional groups has opened new avenues to design high surface area catalyst supports and materials with high photochemical and electrochemical activity [9]. CNT are also among the strongest and stiffest materials yet discovered in terms of tensile strength and elastic modulus. However, the rod-like shape of certain types of CNT has been compared to asbestos fibers, raising concern that their widespread use may lead to serious health consequences. It has been previously reported that rigid rod-like CNT are able to induce asbestos like pathogenicity when introduced as a single intraperitoneal dose to mice, acting as a surrogate tissue for mesothelial lining of the lung [10]. Moreover, subcutaneous [11], intratracheal [12] and intranasal [11] administration of multi-walled CNT have been shown to aggravate allergen-induced airway inflammation and inhalation of high-dose of the CNT has been found to reach the sub-pleural tissue and induce sub-pleural fibrosis in OVA-sensitized mice [13]. In the present study, we examined whether inhalation exposure, mimicking real-life exposure scenarios, to rigid rod-shaped CNT (rCNT) or to tangled CNT (tCNT) without pre-sensitization to any allergens is able to induce asthma-like pathologies.

## Results

### Inhaled rCNT trigger eosinophilia in lungs

**Figure 1:**
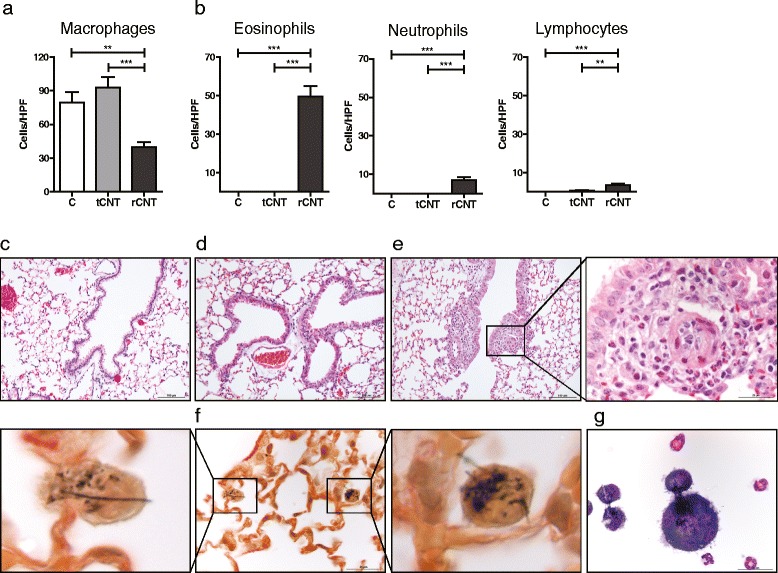
**Inhaled rCNTs induce strong pulmonary eosinophilia in mice after exposure for 4 consecutive days.** C57BL/6 mice were exposed to rCNT or tCNT for 4 h/day for 4 consecutive days and the samples were collected 24 h after the last treatment. **a**,**b**: BAL cell counts showed the ability of rCNT to cause a decrease in the number of alveolar macrophages **(a)** and induce recruitment of inflammatory cells, especially eosinophils **(b)**. **c**-**e**: H&E-stained lung sections of untreated **(c)**, tCNT-treated **(d)** and a rCNT-treated **(e)** mouse exhibiting that eosinophilia was specific to rCNT (e, see inset for eosinophils). **f**: internalized rCNT fibers in alveolar macrophages were detected in interstitial areas in Picrosirius red-stained lung sections. **g**: an image of BAL cells showing a foreign-body giant cell containing rCNT, macrophages undergoing “frustrated phagocytosis”, eosinophils and a neutrophil. Columns and error bars in a and b represent mean values ± standard error of mean (SEM; n = 9-20). Images c and d are shown with a 100 μm scale bar, f-g and the inset is shown with a 25 μm scale bar. **P < 0.01; ***P < 0.001. C, untreated control group; HPF, high power field.

To study whether CNT have the ability to recruit eosinophils in the airways, a distinctive sign of allergic airway inflammation, mice were exposed to rCNT or tCNT aerosol for 4 h/day for 4 consecutive days. rCNT caused a decrease in the number of alveolar macrophages in bronchoalveolar lavage fluid (BAL; Figure 1a) and induced the recruitment of inflammatory cells, especially eosinophils (Figure 1b), while no significant changes in influx of these cell types in response to tCNT treatment were found. Likewise, in the lung tissue, the presence of eosinophils was seen after rCNT treatment, but not after tCNT (Figure 1c-e). Moreover, rCNT fibers were detected in alveolar macrophages in interstitial areas (Figure 1f) and macrophages were found to undergo “frustrated phagocytosis” and form foreign-body giant cells specifically in response to rCNT (Figure 1g). These results indicate that unlike inhaled tCNT, rCNT are able to trigger eosinophilic inflammation in lungs.

### Inhalation of rCNT induces signs associated with allergic airway inflammation

**Figure 2:**
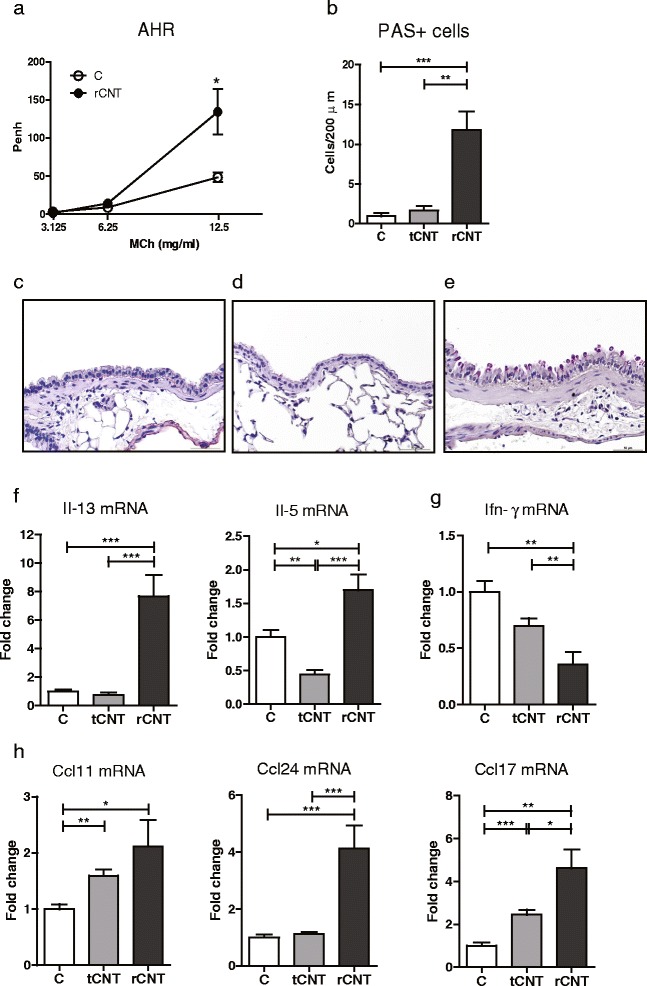
**Inhalation to rCNT induces AHR, mucus secretion and induction of Th2 type cytokines in the lungs. a**: BALB/c mice exposed to rCNT for 4 h/day on 4 consecutive days showed enhanced AHR to metacholine on day 5. **b**-**h**: using the same exposure schedule, C57BL/6 mice were exposed to tCNT or rCNT and sacrificed on the following day. **b**: PAS-stained lung sections were used to determine the numbers of mucin-producing goblet cells. **c**-**e**: representative images of PAS-stained lung tissue of an untreated mouse **(c)** and mice exposed to tCNT **(d)** and rCNT **(e)** that show markable activation of mucin-producing cells in lungs of rCNT-exposed but not in tCNT-exposed mice. **f**, **g**: the mRNA expression levels measured in lung tissue revealed an upregulation of Th2 type cytokines Il-13 and Il-5 **(f)** and downregulation of Th1 cytokine Ifn-γ **(g)** after exposure to rCNT. **h**: furthermore, mRNA expression of eosinophil-attracting chemokines Ccl11, Ccl24 and Ccl17 was triggered in lungs by rCNT. Results of AHR measurements **(a)** are expressed as mean enhanced pause values (Penh) ± SEM (n = 9-11). PAS + cell counts **(b)** represent the average of positive cells per 200 μm of bronchus surface counted from three bronchi per mouse in control group and six bronchi per mouse in CNT-exposed groups (n = 10-20). Images c-e are shown at x400 magnification with a 50 μm scale bar. mRNA expression levels measured by qRT-PCR are presented as fold changes compared to untreated control mice (n = 10-20). *P < 0.05; **P < 0.01; ***P < 0.001. MCh, metacoline; C, control group.

Classical signs of asthma include mucus hypersecretion, AHR and expression of Th2 type cytokines. Since it is well known that AHR cannot be elicited in the C57BL/6 mouse strain [14], we used BALB/c mice to demonstrate that rCNT inhalation induces AHR to inhaled metacholine (Figure 2a; Cellular infiltration in BAL and cytokine/chemokine expression in the lung tissue of rCNT exposed BALB/c mice is given in Additional file 1). We also found that rCNT, but not tCNT, triggered significant activation of mucin-producing goblet cells 24 h after 4-day inhalation exposure in C57BL/6 mice (Figure 2b-e). In addition, up-regulation of Th2 type cytokines Il-13 and Il-5 transcriptional levels (Figure 2f) and down-regulation of Th1 cytokine Ifn-γ (Figure 2g) after exposure to rCNT were evidenced in lung tissue. Furthermore, mRNA expression of eosinophil-attracting chemokines Ccl11, Ccl24 and Ccl17 was triggered by rCNT (Figure 2h). tCNT treatment had no effect on the expression of Il-13, Ifn-γ and Ccl24, however, it suppressed Il-5 and elevated Ccl11 and Ccl17 production. These results show that inhaled rCNT cause AHR, mucus production and trigger production of Th2 type cytokines and eosinophil chemoattractants, and thereby suggest that rCNT fibers can elicit a condition similar to allergic airway inflammation.

### Mast cells partially regulate the development of rCNT-triggered allergic-like airway inflammation

**Figure 3:**
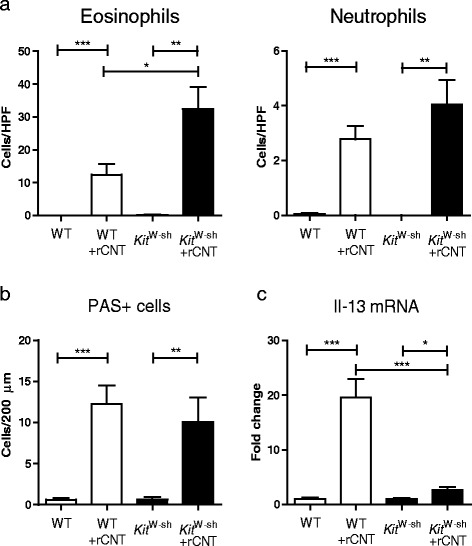
**Mast cells play a partial role in the development of rCNT-triggered asthma.** Wild type (WT) C57BL/6 mice and mast cell deficient *Kit* ^W-sh^ mice in C57BL/6 background were exposed to rCNT for 4 h/day on 4 consecutive days and were sacrificed on day 5. **a**: BAL cell counts showed that mast cells affect the migration of eosinophils but not neutrophils into the lungs. **b**: the numbers of PAS^+^ cells did not differ in WT and *Kit* ^W-sh^ mice indicating that mast cells do not regulate the activation of goblet cells. **c**: the mRNA expression levels measured in lung tissue revealed a significant down-regulation of Th2 type cytokine Il-13 in *Kit* ^W-sh^ mice compared with WT mice. PAS^+^ cell counts **(b)** represent the average of positive cells per 200 μm of bronchus surface counted from three bronchi per mouse in control group and six bronchi per mouse in CNT-exposed groups (n = 7-9). mRNA expression levels in c are presented as fold changes relative to untreated control mice of the corresponding strain (n = 7-9). *P < 0.05; **P < 0.01; ***P < 0.001.

Mast cells are one of the main mediators in immediate allergic reactions. To explore the involvement of this cell type in rCNT-induced inflammation, mast cell deficient *Kit*^*W*-sh^ mice were used. 24 h after 4-day inhalation exposure to rCNT, significantly elevated number of eosinophils was found in *Kit*^*W*-sh^ as compared to wild type (WT) C57BL/6 mice, however, no significant differences were seen in the number of neutrophils (Figure 3a) and lymphocytes (Additional file 2a). Mast cells were not found to mediate the activation of goblet cells as the number of periodic acid-Schiff (PAS)^+^ cells were comparable between both strains after rCNT exposure (Figure 3b). While rCNT-induced mRNA expression of Il-13 was highly dependent on mast cells (Figure 3c), production of Ccl11, Ccl24 and Ccl17 was not significantly affected by these cells (Additional file 2b).

### Transcriptome analysis of lung tissue reveals rapid activation of innate immune system in response to inhaled rCNT

**Figure 4:**
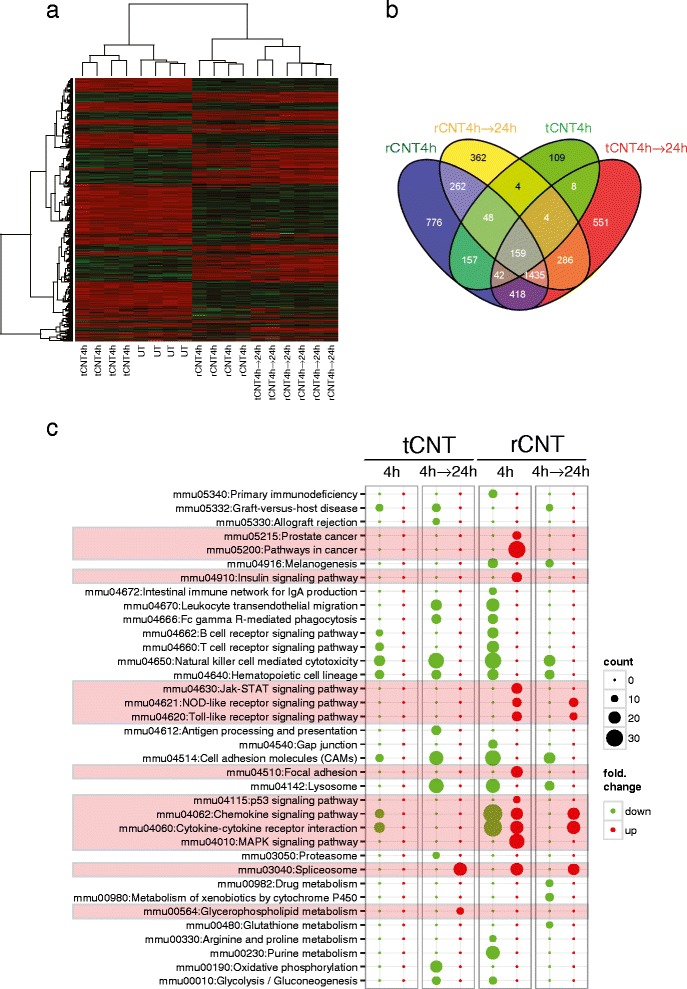
**Transcriptome analysis of lung tissue reveals rapid activation of innate immune system in response to inhaled rCNT. a**: heatmap of differentially expressed genes (linear FC > |1.5|, post hoc adjusted P-value = 0.01) in lungs of mice exposed to rCNT or tCNT for 4 h and sacrificed either immediately after exposure or on the following day. Genes are arranged by hierarchical clustering analysis. Untreated (UT) and tCNT-exposed lung samples share similar expression patterns shortly after 4-h exposure. Red color indicates higher expression while green denotes lower expression. **b**: Venn diagram of the microarray results summarizing the numbers of differentially expressed genes (linear FC > |1.5|, post hoc adjusted P-value = 0.01) in lung tissue after 4 h exposure to CNT. The diagram shows that rCNT cause a markable expression of multiple distinct genes already after 4-h exposure (776 genes) while the number of genes specific to tCNT is notably lower (109 genes). **c**: pathway bubble-plot shows that inhalation of rCNT activates several innate immunity-associated pathways after the 4 h exposure. The diameter of each circle represents the number of genes annotated in each pathway (legend “count”). Red color indicates up-regulated pathways and green color down-regulated pathways compared to untreated controls.

We further investigated the early regulatory events at the transcriptomic level (NCBI GEO Accession Number GSE50176, Additional file 3). C57BL/6 mice were hence exposed by inhalation to rCNT or tCNT once for 4 h, and sacrificed either immediately or 24 h after the cessation of the exposure. At 4 h, the tCNT treatment had a relatively mild effect on the gene expression and these samples tended to cluster together with the untreated ones (Figure 4a). On the contrary, the effect of rCNT was remarkable at the same time point, impacting many more genes and with much greater amplitude of variation from the baseline samples (Figure 4a,b). At 4 h, rCNT exposure induced almost exclusively pathways essential for the innate immunity (e.g. NOD-like receptor and Toll-like receptor signaling pathways, Chemokine signaling pathways and Cytokine-cytokine receptor interaction pathways) (Figure 4c). Moreover, cancer related pathways as well as insulin signaling pathways were up-regulated after rCNT exposure. In sharp contrast, the innate immunity pathways were either not activated or were down-regulated after a 4 h inhalation of tCNT (Figure 4c). At 24 h following the 4 h exposure, a stronger effect of tCNT took place on a wider set of genes, causing changes similar in size and range of variation to those elicited by rCNT. Nonetheless, the gene expression signatures of rCNT and tCNT were quite distinct, as the group of the genes with similar behavior in both treatments was relatively small (Figure 4b, Additional file 4). In addition, direct comparison of the transcriptome of rCNT and tCNT exposed mice at each time point revealed that innate immunity pathways and chemokine-cytokine signaling pathways were significantly more expressed after rCNT, whereas lysosomal activity and carbohydrate metabolism pathways were induced by tCNT (Additional file 5). Some highly significant genes in the microarray analysis also relevant to asthma (Ccl2, Ccl11, Ccl24 and Cxcl5) were further confirmed by quantitative PCR (Additional file 6).

### Alveolar macrophages and mast cells play a role in the initiation of the inflammatory and allergy-like rCNT-induced responses

**Figure 5:**
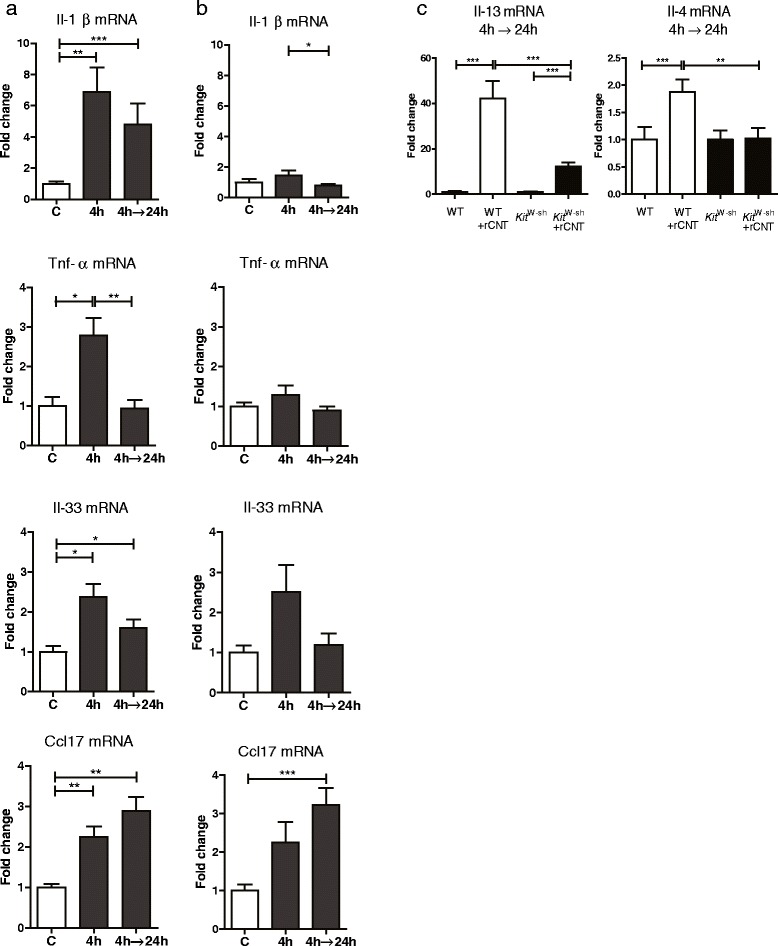
**Alveolar macrophages and mast cells mediate the early inflammatory and pro-allergic responses induced by rCNT. a**,**b**: BAL and lung tissue were collected from C57BL/6 mice exposed to rCNT for 4 h and sacrificed either immediately or on the following day. Comparison of mRNA expression levels of cytokines/chemokines in BAL cells **(a)** and lung tissue **(b)** showing that pro-inflammatory cytokines Il-1β and Tnf-α are produced by alveolar macrophages which are the major cell type in BAL at early time points (data not shown) while Th2-promoting cytokine Il-33, and eosinophil-attracting chemokine Ccl17 are expressed in BAL cells as well as in other cells types present in lung tissue. **c**: levels of Th2 type cytokines Il-13 and Il-4 in lungs were dependent on the presence of mast cells as the expression of these cytokines was significantly lower in *Kit* ^W-sh^ mice exposed to rCNT for 4 h and sacrificed on the next day. mRNA expression levels in a and b are presented as fold changes relative to untreated control mice (n = 5-8). In c, values indicate fold changes compared with control mice of the corresponding strain (n = 7-9). *P < 0.05; **P < 0.01; ***P < 0.001. C, untreated control group.

To investigate the involvement of macrophages in the early stages of rCNT-triggered reactions, we exposed C57BL/6 mice for 4 h and collected BAL and lung tissue samples immediately or 24 h after the exposure. Since macrophages are the major cell type in BAL at early stages of the inflammatory response, we compared the mRNA expression of pro-inflammatory and pro-allergic cytokines in BAL cells and lung tissue (Figure 5a,b). We found that Il-1β and Tnf-α are produced mainly by alveolar macrophages while Th2-promoting cytokine Il-33, and eosinophil-attracting chemokine Ccl17 are expressed in BAL cells as well as in other cell types present in lung tissue. In addition, we found that BAL macrophages express Ccl2 and Ccl7, which may contribute to the stimulation of monocyte recruitment to the site of inflammation (Additional file 7).

The role of mast cells was investigated in lung tissue of C57BL/6 and *Kit*^W-sh^ mice exposed to rCNT for 4 h and sacrificed on the following day. Results of mRNA expression levels of Th2 type cytokines and pro-allergic chemokines showed that Il-13 and Il-4 were derived from mast cells (Figure 5c) while expression of Il-33, Il-5, Ccl11, Ccl24 and Ccl17 was not influenced by deficiency of these cells (Additional file 8).

## Discussion

Along with the increase in production and use of CNT in numerous applications, also occupational and environmental exposure to these materials has increased. This has raised awareness of the potential harmfulness of CNT on human health. One of the main exposure routes associated with ENM is the respiratory tract and hence most of the *in vivo* studies have concentrated on investigating pulmonary effects.

Several studies have shown that CNT fibers have adjuvant capacity as they aggravate allergen-induced airway inflammation [11-13,15,16]. Here, we investigated whether CNT have the ability to induce characteristics similar to allergic airway inflammation in healthy mice. Using different types of CNT, we exposed C57BL/6 mice to an aerosol of rCNT or tCNT repeatedly for 4 h, for a total of 4 days and collected samples 24 h after the last treatment, thus mimicking a one-week occupational exposure. Inhalation of rCNT elicited a drastic infiltration of eosinophils but only a minor increase in neutrophils. Pulmonary eosinophilia is a classical sign of allergic airway inflammation and asthma in which eosinophils are critically involved in the induction of airway hyperreactivity, elevated mucus production, airway remodeling and asthma exacerbations [17,18]. In contrast, exposure to tCNT did not cause morphologically evident lung inflammation. In a study by Park *et al.* neutrophilic inflammation was induced one day after intratracheal administration of multi-walled carbon nanotubes (MWCNT) [19]. Similarly, Morimoto *et al.* reported MWCNT-induced neutrophilia peaking at day 3 after intratracheal instillation [20]. However, other studies performed *via* pharyngeal aspiration have shown that MWCNT induce an influx of both neutrophils and eosinophils, [21] especially at lower doses [22]. Although the inhalation method is the closest to real-life scenarios, the number of such studies is very limited. In contrast to our observations, a 3-month inhalation of MWCNT induced mild neutrophilic, but not eosinophilic, pulmonary inflammation in rats [23]. Similar phenomenon was seen also in mice where the authors reported pulmonary neutrophilia after a 2-day exposure [24]. Nonetheless, assessing health effects of CNT *in vivo* is complex and different results difficult to compare, due to varying methods of exposure in different organisms as well as the different physico-chemical properties of the tested materials.

In the present study, the rCNT triggered cytopathology included a substantial number of macrophages that attempted to engulf CNT aggregates and a presence of foreign body giant cells (FBGC). However, the FBGC did not appear in mice after tCNT treatment. Macrophages undergoing “frustrated phagocytosis” and formation of FBGC in response to MWCNT have been reported earlier after intraperitoneal injection using the same material as in our study [10]. Mangum *et al.* [25] found carbon bridges between macrophages after a single oropharyngeal aspiration of SWCNT in rats. Giant cells arise from a fusion of macrophages in response to large foreign material [26]. These cells are recognized as the pathological hallmark of granulomatous diseases [26]. Although we did not observe granulomas after the 4-day exposure, FBGC might be an indication of their upcoming formation. Their formation has been induced by alternative activation of macrophages *in vitro* by stimulation of Th2 type cytokines Il-4 and Il-13 [27,28]. Alternatively activated macrophages have been also associated with allergic airway inflammation [29].

Excessive mucus secretion in the airways and AHR are classical features of allergic airway inflammation [2]. We found that pulmonary eosinophilia caused by rCNT was accompanied by increased goblet cell hyperplasia as well as elevation of AHR to inhaled metacholine. Moreover, the up-regulation of Th2 type cytokines Il-13 and Il-5, and down-regulation of Th1 type cytokine Ifn-γ in the lung tissue after rCNT inhalation supports the induction of allergic-like airway inflammation. Th2 type cytokines are typically found in the asthmatic airways [1] and the decreased level of Ifn-γ is to be expected since Th1 type cytokines are considered to be counter-regulatory to the Th2 type cytokines. Finally, eosinophil-chemoattractants were notably elevated, supporting the rCNT-induced pulmonary eosinophilia. To our knowledge, no previous studies exist describing an array of features characteristic to allergic airway inflammation and asthma after bare inhalation exposure to CNT, as seen in the present study. It should be also noted that full-blown allergic-like inflammation was elicited just in 5 days and without the conventional protein allergen or treatment with an artificial adjuvant substances (e.g. aluminum hydroxide). The current paradigm proposes that asthma is initiated by a sensitization phase, during which contact with (protein) allergens alerts the immune system, polarizes allergen-specific Th2 lymphocytes and induces secretion of allergen specific-IgE antibodies [2]. In our experimental set up, the conventional sensitization phase cannot be developed due to the very short period of time and the lack of protein allergens, indicating that polarization of Th2 lymphocytes and generation of IgE antibodies is not possible. Thus, the rCNT-induced allergic-like inflammation is likely caused by the activation of the innate immune system, eliciting a novel type of allergic-like airway inflammation in the occupational health context.

The Reactive Airways Dysfunction Syndrome (RADS) is an occupational asthma-like syndrome that develops rapidly after a single exposure to high levels of irritating fume, smoke, vapor or aerosol [30]. RADS differs from traditional asthma especially due to its acute nature with no latency period. Initial respiratory symptoms are followed by asthma-like symptoms and airway hyperresponsiveness. Irritant-induced asthma (IrIA) is also used to describe an asthmatic syndrome that results from a single or multiple high dose exposures to irritant products. Interestingly, both RADS and IrIA induce significant upper airway symptoms, nonspecific inflammation, neurogenic inflammation, primarily lymphocytic cellular infiltrate, macrophage activation, mast cell degranulation and epithelial desquamation [31-34]. The airway inflammation observed in the present study seems to be something in between the classic asthma and IrIA. Even though the inflammation lacks the sensitization phase and protein allergens, it still possesses classical features of allergic asthma such as Th2 cytokines, mucus production and pulmonary eosinophilia. Rather than allergens, the rCNT seem to act more like irritants in inducing a fast and dramatic innate immunity mediated Th2-type airway inflammation similar to allergic asthma.

Mast cells are known to be the central effectors in immediate hypersensitivity reactions [35]. After encountering an allergen, IgE-sensitized mast cells release a broad panel of bioactive mediators and Th2 type cytokines (e.g. IL-4, IL-5, IL-13) in order to initiate and promote airway inflammation, and to strongly induce development of T cell responses [3,35,36]. Although traditional protein allergen-sensitization was not used in our experimental setting, we were interested in exploring whether the mast cells would also mediate the rCNT-driven inflammation. We found that the influx of eosinophils was increased and the expression of Il-13 was significantly decreased in the lungs of the mast cell deficient mice. However, the goblet cell activation was not dependent on these cells and the eosinophil attracting chemokines were likely derived from other sources. These findings suggest that mast cells modulate the rCNT-induced pulmonary inflammation partially.

Early events in the CNT-induced airway inflammation were examined by transcriptomics profiling. The analysis revealed that shortly after the 4 h exposure, the tissue of untreated and tCNT-exposed mice shared similar expression patterns, which were drastically distinct from rCNT-treated mice. Moreover, only rCNT induced increased expression of a number of inflammation-associated pathways. Since the transcriptomics data suggested that rCNT activate several innate immunity pathways, we wanted to estimate the role of alveolar macrophages and lung tissue cells in these early events. BAL cells were isolated immediately after and 24 h following the 4 h rCNT exposure and mRNA expression levels of BAL cells were compared to the mRNA levels of lung tissue of the same animals. Most of the pro-inflammatory Il-1β and Tnf-α expression was likely derived from the rCNT activated alveolar macrophages (i.e. BAL cells) as the tissue expression levels were much lower. On the other hand, the pro-allergic Il-33 [37], which promotes the activation of mast cells [38] and alternative activation of macrophages, was expressed by alveolar macrophages but also by other resident tissue cells. Similarly, the pro-allergic chemokine Ccl17 [39], whose overexpression has been observed earlier in lung macrophages of asthmatic patients [40], was highly up-regulated in BAL macrophages and lung tissue. Taken together, the present results demonstrate that rCNT activated alveolar macrophages are an important source of not only proinflammatory cytokines but also allergy promoting cytokines and chemokines. To our knowledge the transcriptomics analysis is a rather rarely used approach in similar studies and the early events in the CNT exposed mice appear weakly documented.

No significant induction of Il-13 and Il-4 was observed from the isolated BAL cells (data not shown) after a 4 h exposure to rCNT, suggesting that the alveolar macrophages are not synthetizing these Th2 cytokines at the early stages. Since mast cells are an important source of Th2 type cytokines, [35] we investigated their potential role as an early source of these cytokines. By using mast cell deficient mice we showed that the expression of Th2 type cytokines Il-13 and Il-4 is significantly decreased in the absence of mast cells suggesting that mast cells are likely an early source of these Th2 cytokines in rCNT induced inflammation. This is also supported by Katwa *et al.* [41] that report MWCNT aspiration which actuates the extracellular release of Il-33 in lung tissue, leading to the activation of mast cells through the ST2 receptor and causing adverse pulmonary and cardiovascular responses. It has been previously reported that MWCNT induced Il-33 secretion from airway epithelia activating innate lymphoid cells and their release of Il-13 and Il-5 [21]. In order to evaluate the possible role of innate lymphoid cells in the present study we measured innate lymphoid cell 2 (ILC2) markers Il-25 and ROR-α [42] in both BAL cells and lung tissue, but found no up-regulation in their expression in response to rCNT treatment (data not shown). Therefore, we suggest that the early events of MWCNT-induced responses include the activation of mast cells by Il-33 derived from macrophages or non-immune lung cells [41]. However we cannot completely exclude the role of ILC2 as their functions and markers are to date not completely clear [43]. It is of interest that carbon materials, in this case carboxy-fullerenes, can also act as inhibitors of allergic reactions by affecting the mast cell function [44].

In summary, we demonstrate that inhalation of rigid rod-shaped CNT induces all the signs of allergic airway inflammation. Experiments with mast cell deficient mice demonstrated that mast cells mediate the rCNT induced pulmonary eosinophilia and the expression of Il-13. Exploration of the early events by transcriptomics analysis reveals that a 4 h exposure to rCNT causes dramatic up-regulation of genes involved in innate immunity and cytokine/chemokine pathways, which also explains the pulmonary inflammation seen after one-week exposure. Early pro-inflammatory cytokines as well as pro-allergic cytokines and chemokines are synthetized by rCNT-activated alveolar macrophages, while mast cells are likely an early source of rCNT induced Il-4 and Il-13.

Our results indicate that in addition to the previously described asbestos associated pathologies, inhalation of rod-like CNT is able to induce a novel innate immunity mediated allergic-like airway inflammation-like reaction. They also highlight marked dissimilarities in the ability of different CNT to impact health. These observations should be taken into account in the risk assessment as well as in safety and protection measures when exposure to CNT is possible. However more knowledge on the long term effects, clinical impacts and dose–response relationships is still needed to draw solid conclusions on the risks of CNT.

## Methods

### CNT and their characterization

**Figure 6:**
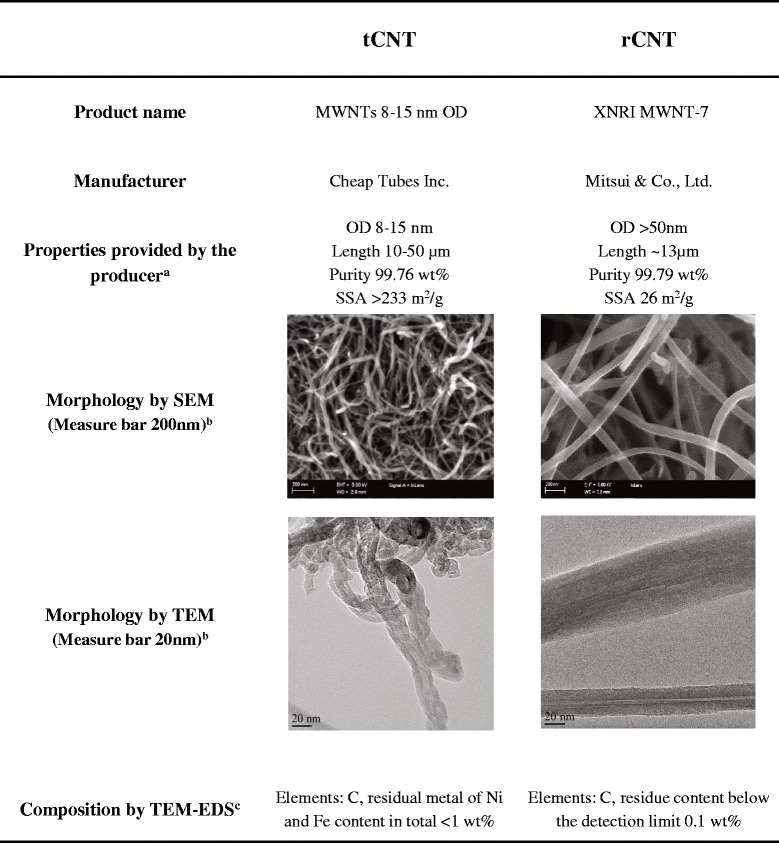
**Table of characteristics of the used multi-walled carbon nanotubes.** ^a^Size, purity and specific surface area are reported in the table, as provided by the vendor. ^b^Morphology of the materials is pictured by electron microscopy (SEM and TEM). ^c^Elemental composition shown is the average of three separate analyses. CNT, carbon nanotubes; EDS, energy dispersive X-ray spectroscopy; OD, outside diameter; SEM, scanning electron microscopy; SSA, specific surface area; TEM, transmission electron microscopy; wt%, weight percent.

The types of MWCNT used in the study included a sample of rod-like MWCNT (rCNT; Mitsui & Co., Ltd., Tokyo, Japan) that had straight, inflexible and rigid fibers, and a sample of tangled MWCNT (tCNT; Cheap Tubes, Inc., Brattleboro, VT, USA) which consisted of curved fibers. The morphology of the CNT was characterized by electron microscopy (Zeiss ULTRAPlus FEG-SEM, Carl Zeiss NTS GmbH, Oberkochen, Germany; Jeol JEM 2010 TEM, Jeol Ltd., Tokyo, Japan) and their composition was analyzed by X-ray energy dispersive spectroscope (EDS ThermoNoran Vantage, Thermo Scientific, Breda, The Netherlands) attached to Jeol JEM 2010 TEM. Elemental composition data were expressed as the average of three separate analyses. Physico-chemical characteristics of the materials are shown in Figure 6.

### Assessment of bacterial lipopolysaccharide content

Levels of biologically active endotoxin in MWCNT were measured using a kinetic chromogenic Limulus amebocyte lysate assay (Kinetic QCL, Lonza, Walkersville, MD, USA) according to the instructions provided by the manufacturer. Endotoxin levels of the material samples were <0,022 EU/mg.

### Animals

Female C57BL/6, BALB/c and *Kit*^W-sh^/HNihrJaeBsmJ mice (7–8 weeks old) were purchased from Scanbur AB (Sollentuna, Sweden) and quarantined for one week. Mice were housed in groups of four in stainless steel cages bedded with aspen chip and were provided with standard mouse chow diet (Altromin no. 1314 FORTI, Altromin Spezialfutter GmbH & Co., Germany) and tap water *ad libitum* when not in exposure chamber. The environment of the animal room was carefully controlled, with a 12-h dark/light cycle, temperature of 20–21°C, and relative humidity of 40–45%. The experiments were performed in agreement with the European Convention for the Protection of Vertebrate Animals Used for Experimental and Other Scientific Purposes (Strasbourg March 18, 1986, adopted in Finland May 31, 1990). The study was approved by the Animal Experiment Board and the State Provincial Office of Southern Finland.

### Aerosol generation

MWCNT were aerosolized with a fluidized bed aerosol generator (FBAG; TSI Model 3400A [45]) for which the materials were used without any pre-treatment. In the generator, a chain transports CNT to the fluidizing bed where material agglomerates are mechanically broken by 200-μm bronze pellets and continuous air flow. To increase CNT feed to fluidizing bed, additional air flow was used. Since the density of CNT was very low, the material reservoir of the FBAG was not large enough to accommodate material for a whole 4-h exposure. Thus, the reservoir was filled after two hours from the beginning of each exposure. During the experiments, the pressure of the exposure chamber differed from the atmospheric pressure ±200 Pa. Pressure in the chamber was dependent on the experiment flow values and on the air intake, whether it was drawn from room trough HEPA filter or from the generator. Detailed scheme of the inhalation setup is provided in Additional file 9.

### Particle characterization

**Figure 7:**
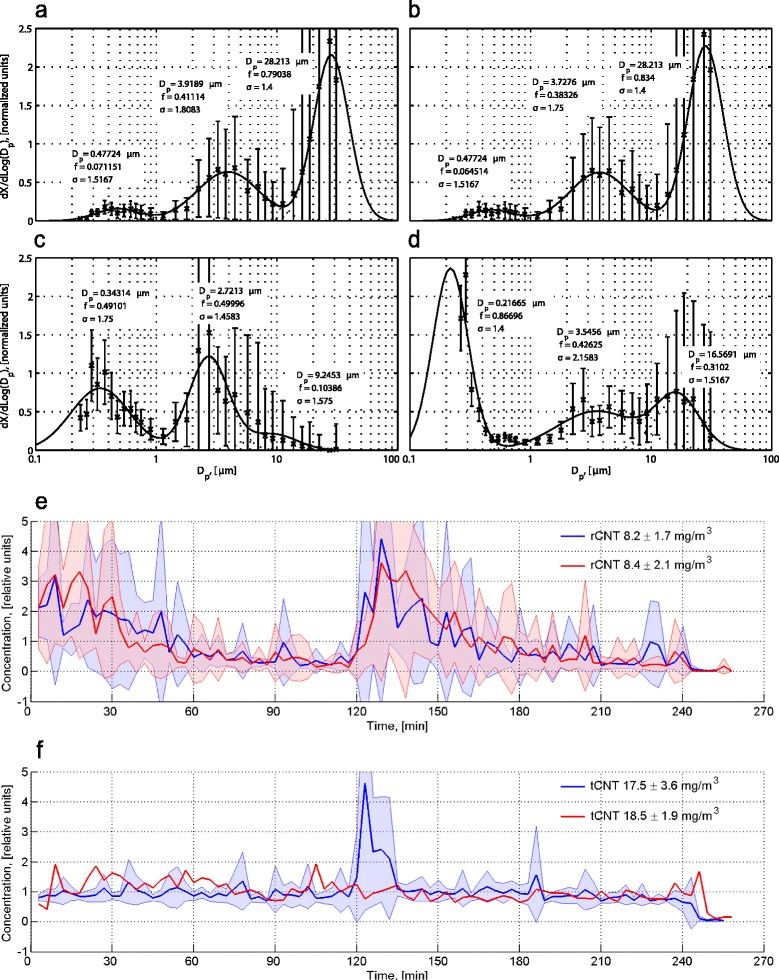
**Concentrations and size distributions of CNT aerosols. a**-**d**: normalized particle size distributions for rCNT experiments with the average concentration of 8.2 mg/m^3^ **(a)** and 8.4 mg/m^3^ **(b)**. Control material (tCNT), with the average particle concentration of 17.5 mg/m^3^ **(c)** and 18.5 mg/m^3^ **(d)**, was used to confirm that rCNT induced inflammatory reaction are truly rCNT-specific and biologically relevant. Parameters of individual log-normal fits: Dp, the mean diameter in relative units; f, the distribution frequency; σ, the geometric standard deviation. **e**,**f**: Particle concentration time series of two representative rCNT **(e)** and tCNT **(f)** experiments. Increase of the concentrations at 120 min is due to refilling of the FBAG reservoir. Bold lines represent means of four 4 h exposures performed on 4 consecutive days and colored areas present standard deviation (SD). In **f**, the red line represents a single 4 h experiment and hence SD is not shown.

Aerosol concentrations were measured with an optical particle sizer (OPS, Grimm Dust Monitor 1.109). We used the instrument calibration refractive index and particle density of 2.6 g/cm^3^. In the Figure 7, the particle size is expressed in relative units and the concentration is shown in normalized units. Gravimetric samples were collected onto nitrocellulose filter (Millipore) and weighted on an analytical scale (RADWAG XA 110/Y; RADWAG Wagi Elektroniczne, Radom, Poland).

### Experimental design

Mice were exposed to aerosolized rCNT or tCNT for 4 hours at a time once or on four consecutive days in a whole-body inhalation chamber. Aerosol mass concentrations used for individual experiments were within a range of 6.2-8.2 mg/m^3^ for rCNT and 17.5-18.5 mg/m^3^ for tCNT. There is still limited amount of information about the concentrations found on workplaces partly due to the challenge of measuring the amounts of airborne CNT. In studies by Erdely *et al.* and Dahm *et al.* [46,47] levels of CNT were found ranging from non-detectable to 1094 μg/m^3^. During CNT synthesis in general, concentrations are most likely in order of micrograms/m^3^ or less, but it is likely that exposure concentrations to large CNT granulates may be high (e.g. milligrams/m^3^) during post-processing of CNT powders. During the experiments, untreated control mice were housed in the same room with CNT-exposed animals. All mice were sacrificed by isoflurane overdose either immediately or 24 h after the exposure(s).

### Measurement of airway responsiveness

Airway responsiveness of BALB/c mice was measured on day 5 using a single chamber, whole-body plethysmograph system (Buxco, Troy, NY, USA) as described earlier [48]. Lung reactivity parameters were expressed as enhanced pause values (Penh). After measurement of lung responsiveness, mice were sacrificed using an overdose of isoflurane and samples were collected for analyses.

### Sample collection

The tracheas of mice were surgically exposed and cannulated with a blunt syringe for collecting the BAL. The lungs were lavaged with 800 μl of Dulbecco’s phosphate buffered saline (DPBS, Thermo Fisher Scientific Inc., Waltham, MA, USA) for 10 s. BAL sample was cytocentrifuged onto a slide, and the cells were stained with May Grünwald-Giemsa (MGG) stain. The remaining cell suspension was centrifuged, supernatant was removed and the cells were fixed in 1:1 ethanol-DPBS mixture. The thoraxes were then opened, and half of the left pulmonary lobe was removed, quick-frozen and stored at −70°C for RNA isolation. The rest of the lung tissue was formalin-fixed, embedded in paraffin, cut, affixed on slides, and stained with hematoxylin and eosin (H&E), PAS and picrosirius red (PSR) solutions.

### BAL cell counts

MGG-stained BAL cells were counted from three high-power fields under light microscope (Leica DM 4000B; Leica, Wetzlar, Germany).

### Histology

Recruitment of inflammatory cells into the lungs and morphological alterations of the tissue were assessed after H&E staining. Lung sections stained with PSR solution were used to evaluate formation of fibrosis but also distribution of CNT within lungs since black fibers were easily detectable on the light background (Figure 1e). The number of mucus-producing cells was determined after PAS-staining from three bronchi per mouse in control group and six bronchi per mouse in treatment groups by counting PAS^+^ cells from 200 μm of bronchus surface under light microscope.

### mRNA expression of cytokines and chemokines in lungs tissue

Lung samples were placed into Lysing matrix D tubes (MP Biomedicals, Illkirch, France) containing 1 ml of TRIsure reagent (Bioline Reagents Ltd., London, UK) and homogenized in a FastPrep FP120 machine (BIO 101, Thermo Savant, Waltham, MA, USA). The RNA extraction was performed following instructions provided by Bioline Reagents. The quantity and purity of isolated RNA was determined by NanoDrop spectrophotometer (ND-1000, Thermo Fisher Scientific Inc., Wilmington, NC, USA). Complementary DNA (cDNA) was synthesized from 500 ng of total RNA in a 25 μl reaction using MultiScribe Reverse Transcriptase and random primers (The High-Capacity cDNA Archive Kit, Applied Biosystems, Foster City, CA, USA) according to the manufacturer’s protocol. The synthesis was performed in a 2720 Thermal Cycler (Applied Biosystems, Carlsbad, CA, USA) starting at 25°C for 10 minutes and continuing at 37°C for 120 minutes. Primers and probes (18S ribosomal RNA, Ccl2, Ccl7, Ccl11, Ccl17, Ccl24, Cxcl5, Ifn-γ, Il-1β, Il-5, Il-13, Il-33, Tnf-α) for real-time quantitative polymerase chain reaction (PCR) analysis were ordered as pre-developed assay reagents from Applied Biosystems. The PCR assays were performed in 96-well optical reaction plates with Relative Quantification 7500 Fast System (7500 Fast Real-Time PCR system, Applied Biosystems) by the manufacturer’s instructions. Amplifications were done in 11 μl reaction volume containing TaqMan universal PCR master mix and primers provided by Applied Biosystems and 1 μl of cDNA sample. Ribosomal 18S was used as an endogenous control.

### DNA microarrays

The microarray data have been deposited in NCBI Gene Expression Omnibus (GEO) database [49] and are accessible through GEO Series accession number GSE50176 (http://www.ncbi.nlm.nih.gov/geo/query/acc.cgi?acc=GSE50176). Total RNA samples isolated from lung tissue as described above were quantified by NanoDrop and the quality was verified by Agilent Bioanalyzer 2100 (Agilent Technologies, Santa Clara, CA, USA). Independent pools of two RNA samples each (total of 600 ng) were labeled using T7 RNA polymerase amplification method (Low Input Quick Amp Labeling Kit, Agilent Technologies), according to the instructions of the manufacturer. cRNAs were then labeled with Cy3 and Cy5 dyes (Agilent Technologies) and hybridized to the Agilent 2-color 60-mer oligo arrays (Agilent SurePrint G3 Mouse GE 8x60K). The slides were washed and scanned with Agilent Microarray Scanner G2505C (Agilent Technologies) and the raw intensity values were obtained with the Feature Extraction software, version 11.0.1.1 (Agilent Technologies). Raw data was quality checked according to the Agilent standard procedures. The median foreground intensities were imported into the R software version 3.0.0 (http://cran.r-project.org) [50] and analyzed with the BioConductor package limma [51]. Log2 transformation and quantile normalization was performed on the single channel data separately, similarly for instance to [52-54] and according to the suggestions by Smyth and Altman [55]. Background correction was not carried out, as suggested by Zahurak et al. [56]. Subsequently the batch effect derived from the labeling was removed using the ComBat method [57] implemented in the sva package [58,59]. The values of the probes recognizing the same NCBI Entrez Gene Ids [60] were further averaged into the final expression matrix. Differentially expressed genes were identified by using linear models and empirical Bayes pairwise comparisons (post hoc adjusted P < 0.01 and linear FC > |1.5|) [61]. The resulting gene sets after Benjamini and Hochberg post hoc correction [57] were considered to be significant and were further studied by the DAVID version 6.7 annotation tool [62] with the default parameters.

### Statistical analysis of cell counts, mRNA levels and AHR

Graphs were constructed and data were analyzed using GraphPad Prism 5 Software (GraphPad Software Inc., San Diego, CA, USA). For all statistical analysis we first performed analysis of variance using one-way ANOVA and when the ANOVA was positive we performed post-testing. An unpaired t-test or Mann–Whitney U-test was used to compare the differences between the groups. A P-value of <0.05 was considered to be statistically significant.

## Additional files

Additional file 1:
**Cellular infiltration in BAL and cytokine/chemokine expression in the lung tissue of rCNT exposed BALB/c mice.** BALB/c mice were exposed to rCNT for 4 h/day on 4 consecutive days and sacrificed on day 5 after BUXCO-measurements. The figure shows (a) cellular infiltration in BAL and (b) proinflammatory cytokine Tnf-α and Il-6 expression in the lung tissue. (c) Th2 cytokine Il-13 and chemokines Ccl11, Ccl17 expression. As can be seen from the figure, in addition to AHR, there are also other strain-specific differences between C57BL/6 and BALB/c described in more detail by e.g. Gueders MM. *et al.* [63] and Watanabe H. *et al.* [64]. mRNA expression levels in b-c are presented as fold changes relative to untreated control mice (n = 5-8). ****P* < 0.001. C, untreated control group; HPF, high power field; rCNT, rod-like multi-walled carbon nanotubes. Additional file 2:
**Recruitment of lymphocytes and expression of** **eosinophil-chemoattractants** **is not regulated by mast cells.** Wild type (WT) C57BL/6 mice and mast cell deficient *Kit* ^W-sh^ mice were exposed to rCNT for 4 h/day on 4 consecutive days and were sacrificed on the following day. a: the number of lymphocytes in BAL. b: mRNA expression levels of eosinophil-attracting chemokines Ccl11, Ccl24 and Ccl17 in lung tissue measured by qRT-PCR. The values indicate fold changes compared with control mice of the corresponding strain (n = 7-9). **P* < 0.05; ***P* < 0.01; ****P* < 0.001. rCNT, rod-like multi-walled carbon nanotubes. Additional file 3:
**Tables of lists of the differentially expressed genes from the microarray experiment.** The tables with the differentially expressed genes in each of the relevant pairwise comparisons are reported in each sheet of the Microsoft Excel archive, named accordingly. In each table, the significantly differentially expressed genes (Benjamini and Hochberg *post hoc* corrected p-value < 0.01 and absolute log2 fold change > 0.58) are ordered according to the decreasing log2 fold change. The NCBI Entrez Gene IDs, gene info and gene symbols are shown. Additionally, for each significant gene, the average expression across the microarray samples, the t-test value, the nominal p-value, the adjusted p-value and the B value are also reported. Additional file 4:
**rCNT exposure causes changes in the number of significant genes at the early stage after exposure.** a: column chart representing the number of up and down regulated genes after 4 h exposure, either sacrificed immediately or the following day. The number of differentially expressed, significant genes (Fold Change > |1.5|, *post-hoc* adjusted*P*-0.01) is highest with rCNT after 4 h (>3000 genes). The amount is already decreasing after 24 h, while the number of expressed genes is on the contrary increasing by time (4 h → 24 h) when exposed to tCNT. b: heatmap indicating the different expression patterns between the rod-like and tangled CNT at 4 h time point, denoting the clear difference between the gene expression patterns after exposure. c: same pattern can be observed on a heatmap showing the differentially expressed genes after 4 h exposure when sacrificed on the following day, separating clearly the rCNT and tCNT from each other. rCNT, rod-like multi-walled carbon nanotubes; tCNT, tangled multi-walled carbon nanotubes. Additional file 5:
**Direct comparison of the transcriptome of rCNT and tCNT exposed mice.** Pathway bubble plot revealed that innate immunity pathways and chemokine-cytokine signaling pathways were significantly more expressed after exposure to rCNT, whereas lysosomal activity and carbohydrate metabolism pathways were induced by exposure to tCNT. The diameter of each circle represents the number of genes annotated in each pathway (legend “count”). Blue color refers to tCNT-induced pathways and brown color to rCNT-induced pathways. rCNT, rod-like multi-walled carbon nanotubes; tCNT, tangled multi-walled carbon nanotubes. Additional file 6:
**qRT-PCR validation of mRNA microarray results.** On the basis of the mRNA microarray results, four genes were selected for validation by qRT-PCR taking into account their biological relevance for allergic asthma. The microarray measurements were considered valid if the expression was concordant with microarray and qRT-PCR p-value was <0,05. qRT-PCR results confirmed significant upregulation of eosinophil-attracting chemokines Ccl11 and Ccl24, monocyte-recruiting chemokine Ccl2 and neutrophil-chemoattractant Cxcl5 in lungs of rCNT-treated mice compared with tCNT-exposed mice at both time points. mRNA expression levels are presented as fold changes relative to untreated control mice (n = 4-8). **P* < 0.05; ***P* < 0.01; ****P* < 0.001. C, untreated control group. rCNT, rod-like multi-walled carbon nanotubes; tCNT, tangled multi-walled carbon nanotubes. Additional file 7:
**During the early stages of inflammation, rCNT stimulate the expression of** **RIG-I-regulated chemokines Ccl2 and Ccl7 in alveolar macrophages.** a, b: C57BL/6 mice were exposed to rCNT for 4 h and sacrificed either immediately or on the following day. RIG-I-regulated chemokines Ccl2 and Ccl7 were significantly expressed at the RNA level in BAL cells (a) and in lung tissue (b) already after 4-h exposure and the expression levels rose even higher by the following day. These data indicate that alveolar macrophages as well as resident lung cells are early source of these chemokines. mRNA expression levels are presented as fold changes compared to untreated control mice (n = 8). **P* < 0.05; ***P* < 0.01; ****P* < 0.001. rCNT, rod-like multi-walled carbon nanotubes. Additional file 8:
**In the early phase of inflammation, mast cells are not the only cell type leading to T helper (Th2) differentiation and eosinophil recruitment in lungs.** Mast cell deficient *Kit* ^W-sh^ mice and their wild type (WT) C57BL/6 control mice and were exposed to rCNT for 4 h and were sacrificed on the following day. a: expression of Il-33 and Il-5 cytokines at the mRNA level in the lungs of rod-like CNT (rCNT)-exposed mice did not differ significantly in *Kit* ^W-sh^ mice compared with WT mice. b: similarly, mRNA expression of Ccl11, Ccl24 and Ccl17 was not dependent on the presence of mast cells. The values represent fold changes compared with control mice of the corresponding strain (n = 7-9). ***P* < 0.01; ****P* < 0.001. rCNT, rod-like multi-walled carbon nanotubes. Additional file 9:
**Scheme of the inhalation system for exposing mice to carbon nanotubes with essential parameters and typical flow values.** In the fluidized bed aerosol generator (FBAG), CNTs are transported to the fluidizing bed with a chain where bronze pellets together with air flow (*Q*_*BF*_) mechanically break material agglomerates. CNT feed to fluidizing bed is enhanced with additional air flow (*Q*_*T*_). Thereafter, aerosolized CNT are directed to whole-body inhalation exposure camber.
