# Supplementary material for: Inhalation of rod-like carbon nanotubes causes unconventional allergic airway inflammation
Source: Part Fibre Toxicol. 2014 Oct 16;11:48. doi: 10.1186/s12989-014-0048-2 (PMC4215016; doi:10.1186/s12989-014-0048-2)
Supplement: Additional file 1: — Cellular infiltration in BAL and cytokine/chemokine expression in the lung tissue of rCNT exposed BALB/c mice. BALB/c mice were exposed to rCNT for 4 h/day on 4 consecutive days and sacrificed on day 5 after BUXCO-measurements. The figure shows (a) cellular infiltration in BAL and (b) proinflammatory cytokine Tnf-α and Il-6 expression in the lung tissue. (c) Th2 cytokine Il-13 and chemokines Ccl11, Ccl17 expression. As can be seen from the figure, in addition to AHR, there are also other strain-specific differences between C57BL/6 and BALB/c described in more detail by e.g. Gueders MM. et al. [63] and Watanabe H. et al. [64]. mRNA expression levels in b-c are presented as fold changes relative to untreated control mice (n = 5-8). ***P < 0.001. C, untreated control group; HPF, high power field; rCNT, rod-like multi-walled carbon nanotubes. [file 12989_2014_48_MOESM1_ESM.pdf]

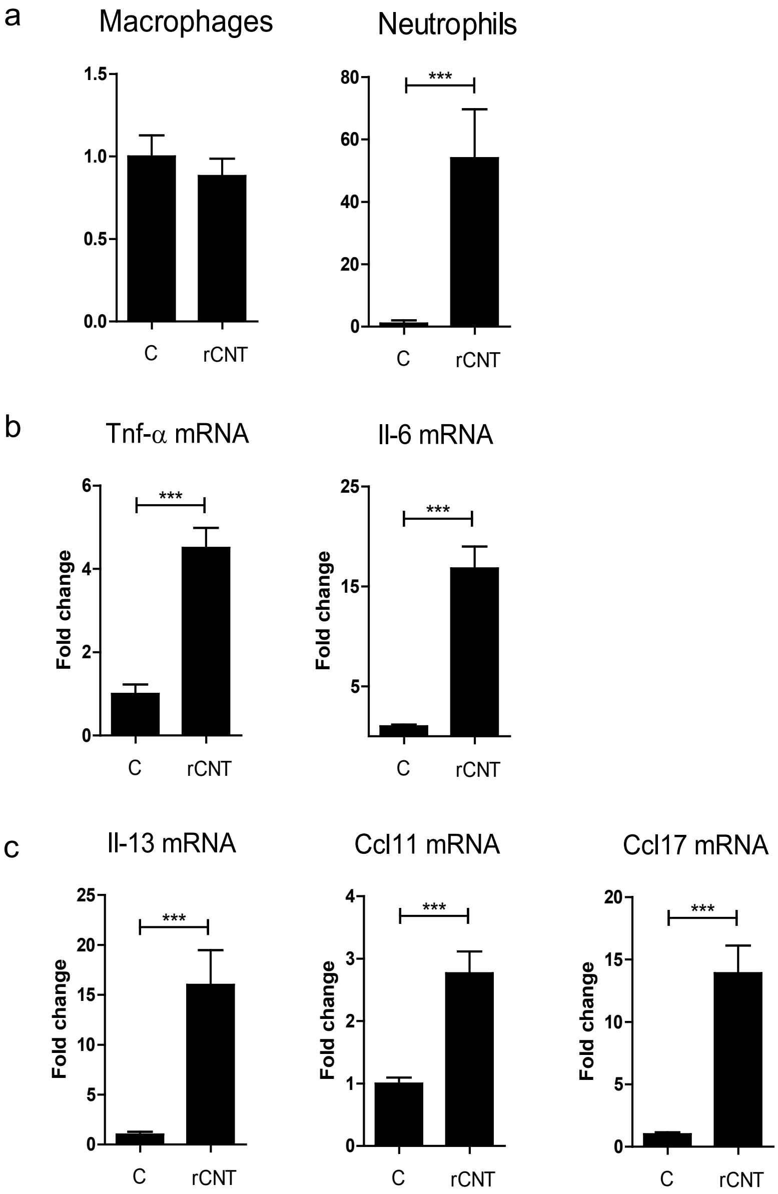

Additional file 1. Cellular infiltration in BAL and cytokine/chemokine expression in the lung tissue of rCNT exposed BALB/c mice.

BALB/c mice were exposed to rCNT for 4 h/day on 4 consecutive days and sacrificed on day 5 after BUXCO-measurements. The figure shows **(a)** cellular infiltration in BAL and **(b)** proinflammatory cytokine Tnf-α and Il-6 expression in the lung tissue. **(c)** Th2 cytokine Il-13 and chemokines Ccl11, Ccl17 expression. As can be seen from the figure, in addition to AHR, there are also other strain-specific differences between C57BL/6 and BALB/c described in more detail by e.g. Gueders MM. *et al.* 2009 and Watanabe H. *et al.* 2004. mRNA expression levels in **b-c** are presented as fold changes relative to untreated control mice (n=5-8). ***P<0.001. C, untreated control group; HPF, high power field; rCNT, rod-like multi-walled carbon nanotubes.
