# Supplementary material for: Inhalation of rod-like carbon nanotubes causes unconventional allergic airway inflammation
Source: Part Fibre Toxicol. 2014 Oct 16;11:48. doi: 10.1186/s12989-014-0048-2 (PMC4215016; doi:10.1186/s12989-014-0048-2)
Supplement: Additional file 2: — Recruitment of lymphocytes and expression of eosinophil-chemoattractants is not regulated by mast cells. Wild type (WT) C57BL/6 mice and mast cell deficient Kit W-sh mice were exposed to rCNT for 4 h/day on 4 consecutive days and were sacrificed on the following day. a: the number of lymphocytes in BAL. b: mRNA expression levels of eosinophil-attracting chemokines Ccl11, Ccl24 and Ccl17 in lung tissue measured by qRT-PCR. The values indicate fold changes compared with control mice of the corresponding strain (n = 7-9). *P < 0.05; **P < 0.01; ***P < 0.001. rCNT, rod-like multi-walled carbon nanotubes. [file 12989_2014_48_MOESM2_ESM.pdf]

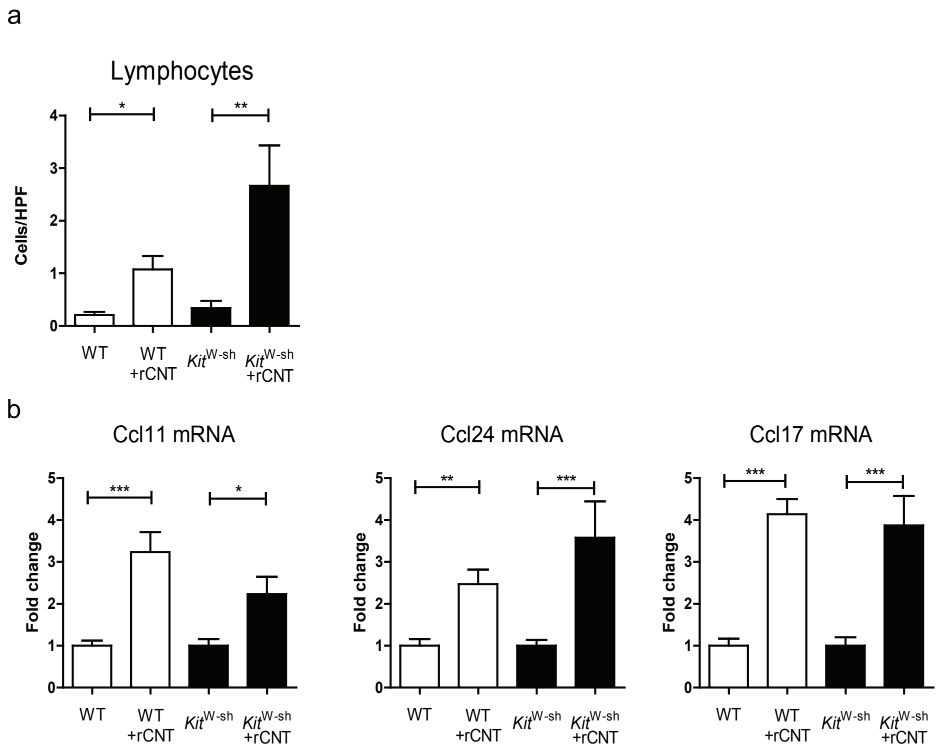

Additional file 2. Recruitment of lymphocytes and expression of eosinophil-chemoattractants is not regulated by mast cells.

Wild type (WT) C57BL/6 mice and mast cell deficient *Kit*$^{W\text{-}sh}$ mice were exposed to rCNT for 4h/day on 4 consecutive days and were sacrificed on the following day. **a:** the number of lymphocytes in BAL. **b:** mRNA expression levels of eosinophil-attracting chemokines Ccl11, Ccl24 and Ccl17 in lung tissue measured by qRT-PCR. The values indicate fold changes compared with control mice of the corresponding strain (n=7-9). *P<0.05; **P<0.01; ***P<0.001. rCNT, rod-like carbon nanotubes.
