# Supplementary material for: Inhalation of rod-like carbon nanotubes causes unconventional allergic airway inflammation
Source: Part Fibre Toxicol. 2014 Oct 16;11:48. doi: 10.1186/s12989-014-0048-2 (PMC4215016; doi:10.1186/s12989-014-0048-2)
Supplement: Additional file 4: — rCNT exposure causes changes in the number of significant genes at the early stage after exposure. a: column chart representing the number of up and down regulated genes after 4 h exposure, either sacrificed immediately or the following day. The number of differentially expressed, significant genes (Fold Change > |1.5|, post-hoc adjustedP-0.01) is highest with rCNT after 4 h (>3000 genes). The amount is already decreasing after 24 h, while the number of expressed genes is on the contrary increasing by time (4 h → 24 h) when exposed to tCNT. b: heatmap indicating the different expression patterns between the rod-like and tangled CNT at 4 h time point, denoting the clear difference between the gene expression patterns after exposure. c: same pattern can be observed on a heatmap showing the differentially expressed genes after 4 h exposure when sacrificed on the following day, separating clearly the rCNT and tCNT from each other. rCNT, rod-like multi-walled carbon nanotubes; tCNT, tangled multi-walled carbon nanotubes. [file 12989_2014_48_MOESM4_ESM.pdf]

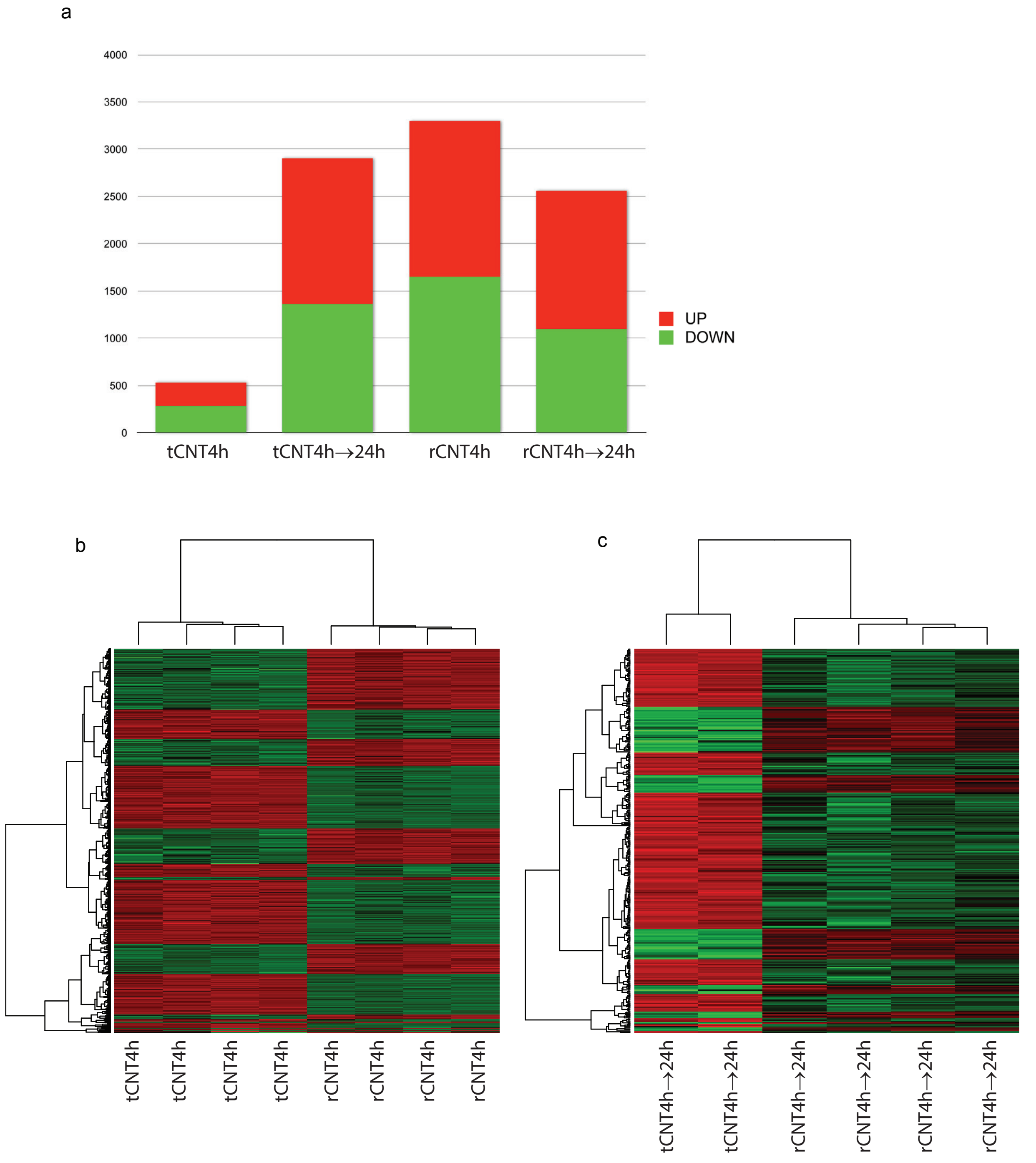

Additional file 4. rCNT exposure causes changes in the number of significant genes at the early stage after exposure.

a: column chart representing the number of up and down regulated genes after 4 h exposure, either sacrificed immediately or the following day. The number of differentially expressed, significant genes (Fold Change >|1.5|, *post-hoc* adjusted*P*-0.01) is highest with rCNT after 4 h (>3000 genes). The amount is already decreasing after 24 h, while the number of expressed genes is on the contrary increasing by time (4h -> 24h) when exposed to tCNT. **b:** heatmap indicating the different expression patterns between the rod-like and tangled CNT at 4 h time point, denoting the clear difference between the gene expression patterns after exposure. **c:** same pattern can be observed on a heatmap showing the differentially expressed genes after 4 h exposure when sacrificed on the following day, separating clearly the rCNT and tCNT from each other. rCNT, rod-like carbon nanotubes; tCNT, tangled carbon nanotubes.
