# Supplementary material for: Inhalation of rod-like carbon nanotubes causes unconventional allergic airway inflammation
Source: Part Fibre Toxicol. 2014 Oct 16;11:48. doi: 10.1186/s12989-014-0048-2 (PMC4215016; doi:10.1186/s12989-014-0048-2)
Supplement: Additional file 5: — Direct comparison of the transcriptome of rCNT and tCNT exposed mice. Pathway bubble plot revealed that innate immunity pathways and chemokine-cytokine signaling pathways were significantly more expressed after exposure to rCNT, whereas lysosomal activity and carbohydrate metabolism pathways were induced by exposure to tCNT. The diameter of each circle represents the number of genes annotated in each pathway (legend “count”). Blue color refers to tCNT-induced pathways and brown color to rCNT-induced pathways. rCNT, rod-like multi-walled carbon nanotubes; tCNT, tangled multi-walled carbon nanotubes. [file 12989_2014_48_MOESM5_ESM.pdf]

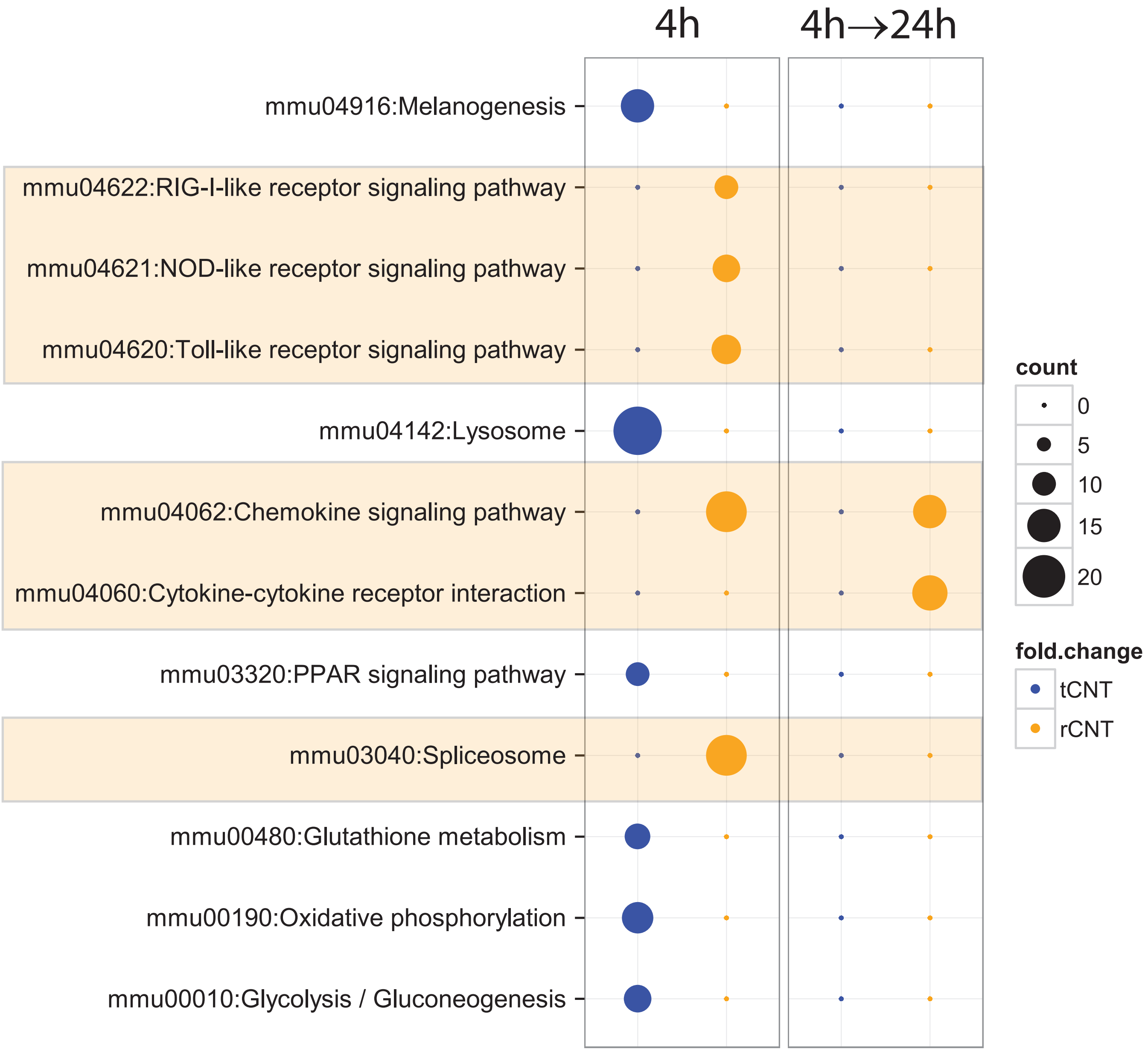

Additional file 5. Direct comparison of the transcriptome of rCNT and tCNT exposed mice.

Pathway bubble plot revealed that innate immunity pathways and chemokine-cytokine signaling pathways were significantly more expressed after exposure to rCNT, whereas lysosomal activity and carbohydrate metabolism pathways were induced by exposure to tCNT. The diameter of each circle represents the number of genes annotated in each pathway (legend "count"). Blue color refers to tCNT-induced pathways and brown color to rCNT-induced pathways. rCNT, rod-like carbon nanotubes; tCNT, tangled carbon nanotubes.
