# Supplementary material for: Inhalation of rod-like carbon nanotubes causes unconventional allergic airway inflammation
Source: Part Fibre Toxicol. 2014 Oct 16;11:48. doi: 10.1186/s12989-014-0048-2 (PMC4215016; doi:10.1186/s12989-014-0048-2)
Supplement: Additional file 6: — qRT-PCR validation of mRNA microarray results. On the basis of the mRNA microarray results, four genes were selected for validation by qRT-PCR taking into account their biological relevance for allergic asthma. The microarray measurements were considered valid if the expression was concordant with microarray and qRT-PCR p-value was <0,05. qRT-PCR results confirmed significant upregulation of eosinophil-attracting chemokines Ccl11 and Ccl24, monocyte-recruiting chemokine Ccl2 and neutrophil-chemoattractant Cxcl5 in lungs of rCNT-treated mice compared with tCNT-exposed mice at both time points. mRNA expression levels are presented as fold changes relative to untreated control mice (n = 4-8). *P < 0.05; **P < 0.01; ***P < 0.001. C, untreated control group. rCNT, rod-like multi-walled carbon nanotubes; tCNT, tangled multi-walled carbon nanotubes. [file 12989_2014_48_MOESM6_ESM.pdf]

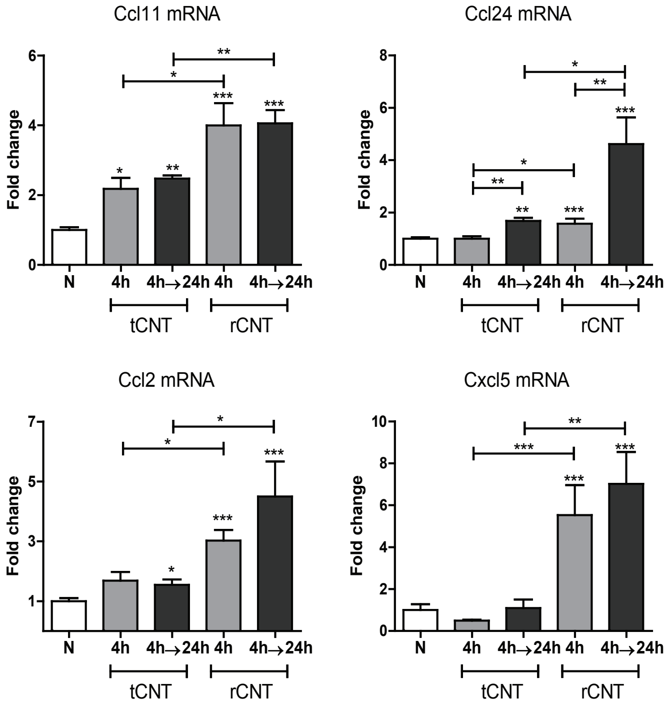

Additional file 6. qRT-PCR validation of mRNA microarray results.

On the basis of the mRNA microarray results, four genes were selected for validation by qRT-PCR taking into account their biological relevance for allergic asthma. The microarray measurements were considered valid if the expression was concordant with microarray and qRT-PCR p-value was <0,05. qRT-PCR results confirmed significant upregulation of eosinophil-attracting chemokines Ccl11 and Ccl24, monocyte-recruiting chemokine Ccl2 and neutrophil-chemoattractant Cxcl5 in lungs of rCNT-treated mice compared with tCNT-exposed mice at both time points. mRNA expression levels are presented as fold changes relative to untreated control mice (n=4-8). *P<0.05; **P<0.01; ***P<0.001. C, untreated control group. rCNT, rod-like carbon nanotubes; tCNT, tangled carbon nanotubes.
