# Supplementary material for: Inhalation of rod-like carbon nanotubes causes unconventional allergic airway inflammation
Source: Part Fibre Toxicol. 2014 Oct 16;11:48. doi: 10.1186/s12989-014-0048-2 (PMC4215016; doi:10.1186/s12989-014-0048-2)
Supplement: Additional file 7: — During the early stages of inflammation, rCNT stimulate the expression of RIG-I-regulated chemokines Ccl2 and Ccl7 in alveolar macrophages. a, b: C57BL/6 mice were exposed to rCNT for 4 h and sacrificed either immediately or on the following day. RIG-I-regulated chemokines Ccl2 and Ccl7 were significantly expressed at the RNA level in BAL cells (a) and in lung tissue (b) already after 4-h exposure and the expression levels rose even higher by the following day. These data indicate that alveolar macrophages as well as resident lung cells are early source of these chemokines. mRNA expression levels are presented as fold changes compared to untreated control mice (n = 8). *P < 0.05; **P < 0.01; ***P < 0.001. rCNT, rod-like multi-walled carbon nanotubes. [file 12989_2014_48_MOESM7_ESM.pdf]

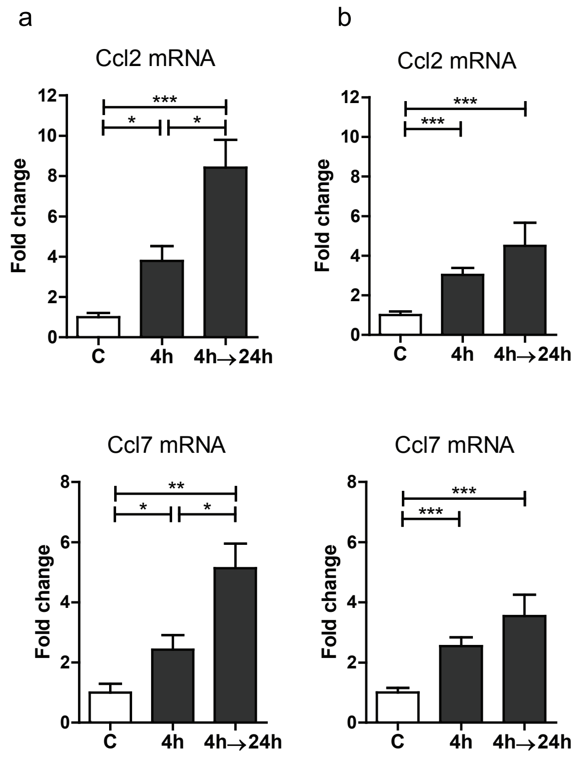

Additional file 7. During the early stages of inflammation, rCNT stimulate the expression of RIG-I-regulated chemokines Ccl2 and Ccl7 in alveolar macrophages.

a, b: C57BL/6 mice were exposed to rCNT for 4h and sacrificed either immediately or on the following day. RIG-I-regulated chemokines Ccl2 and Ccl7 were significantly expressed at the RNA level in BAL cells (**a**) and in lung tissue (**b**) already after 4-h exposure and the expression levels rose even higher by the following day. These data indicate that alveolar macrophages as well as resident lung cells are early source of these chemokines. mRNA expression levels are presented as fold changes compared to untreated control mice (n=8). *P<0.05; **P<0.01; ***P<0.001.
