# Supplementary material for: Inhalation of rod-like carbon nanotubes causes unconventional allergic airway inflammation
Source: Part Fibre Toxicol. 2014 Oct 16;11:48. doi: 10.1186/s12989-014-0048-2 (PMC4215016; doi:10.1186/s12989-014-0048-2)
Supplement: Additional file 8: — In the early phase of inflammation, mast cells are not the only cell type leading to T helper (Th2) differentiation and eosinophil recruitment in lungs. Mast cell deficient Kit W-sh mice and their wild type (WT) C57BL/6 control mice and were exposed to rCNT for 4 h and were sacrificed on the following day. a: expression of Il-33 and Il-5 cytokines at the mRNA level in the lungs of rod-like CNT (rCNT)-exposed mice did not differ significantly in Kit W-sh mice compared with WT mice. b: similarly, mRNA expression of Ccl11, Ccl24 and Ccl17 was not dependent on the presence of mast cells. The values represent fold changes compared with control mice of the corresponding strain (n = 7-9). **P < 0.01; ***P < 0.001. rCNT, rod-like multi-walled carbon nanotubes. [file 12989_2014_48_MOESM8_ESM.pdf]

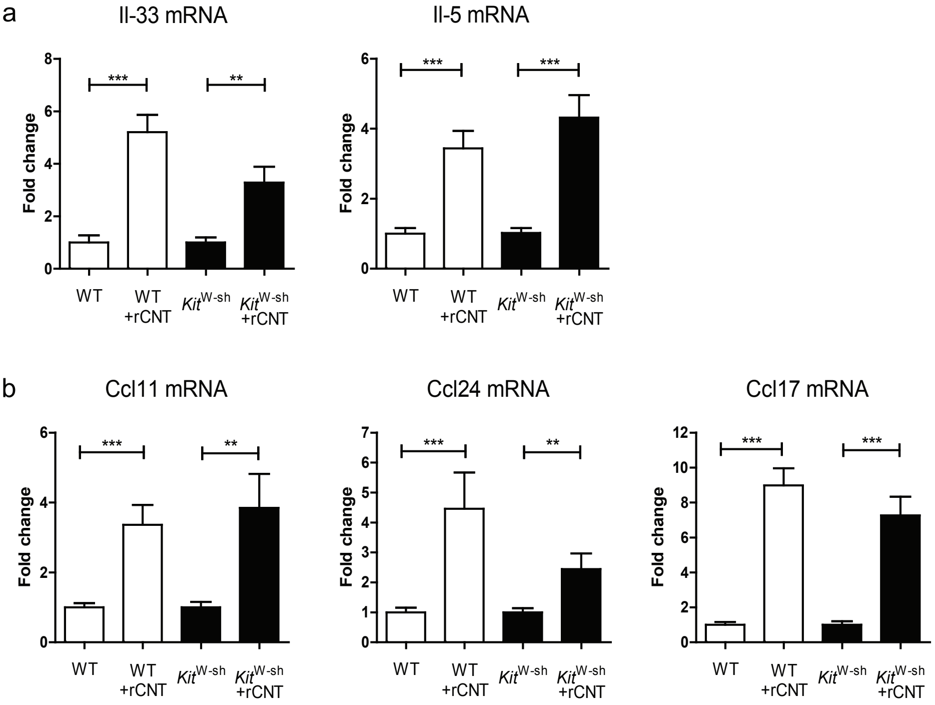

Additional file 8. In the early phase of inflammation, mast cells are not the only cell type leading to eosinophil recruitment in lungs.

Mast cell deficient *Kit*$^{W\text{-}sh}$ mice and their wild type (WT) C57BL/6 control mice and were exposed to rCNT for 4h and were sacrificed on the following day. **a:** expression of Il-33 and Il-5 cytokines at the mRNA level in the lungs of rod-like CNT (rCNT)-exposed mice did not differ significantly in *Kit*$^{W\text{-}sh}$ mice compared with WT mice. **b:** similarly, mRNA expression of Ccl11, Ccl24 and Ccl17 was not dependent on the presence of mast cells. The values represent fold changes compared with control mice of the corresponding strain (n=7-9). $^{**}P<0.01$; $^{***}P<0.001$. rCNT, rod-like carbon nanotubes.
