# Supplementary material for: Inhalation of rod-like carbon nanotubes causes unconventional allergic airway inflammation
Source: Part Fibre Toxicol. 2014 Oct 16;11:48. doi: 10.1186/s12989-014-0048-2 (PMC4215016; doi:10.1186/s12989-014-0048-2)
Supplement: Additional file 9: — Scheme of the inhalation system for exposing mice to carbon nanotubes with essential parameters and typical flow values. In the fluidized bed aerosol generator (FBAG), CNTs are transported to the fluidizing bed with a chain where bronze pellets together with air flow (Q BF) mechanically break material agglomerates. CNT feed to fluidizing bed is enhanced with additional air flow (Q T). Thereafter, aerosolized CNT are directed to whole-body inhalation exposure camber. [file 12989_2014_48_MOESM9_ESM.pdf]

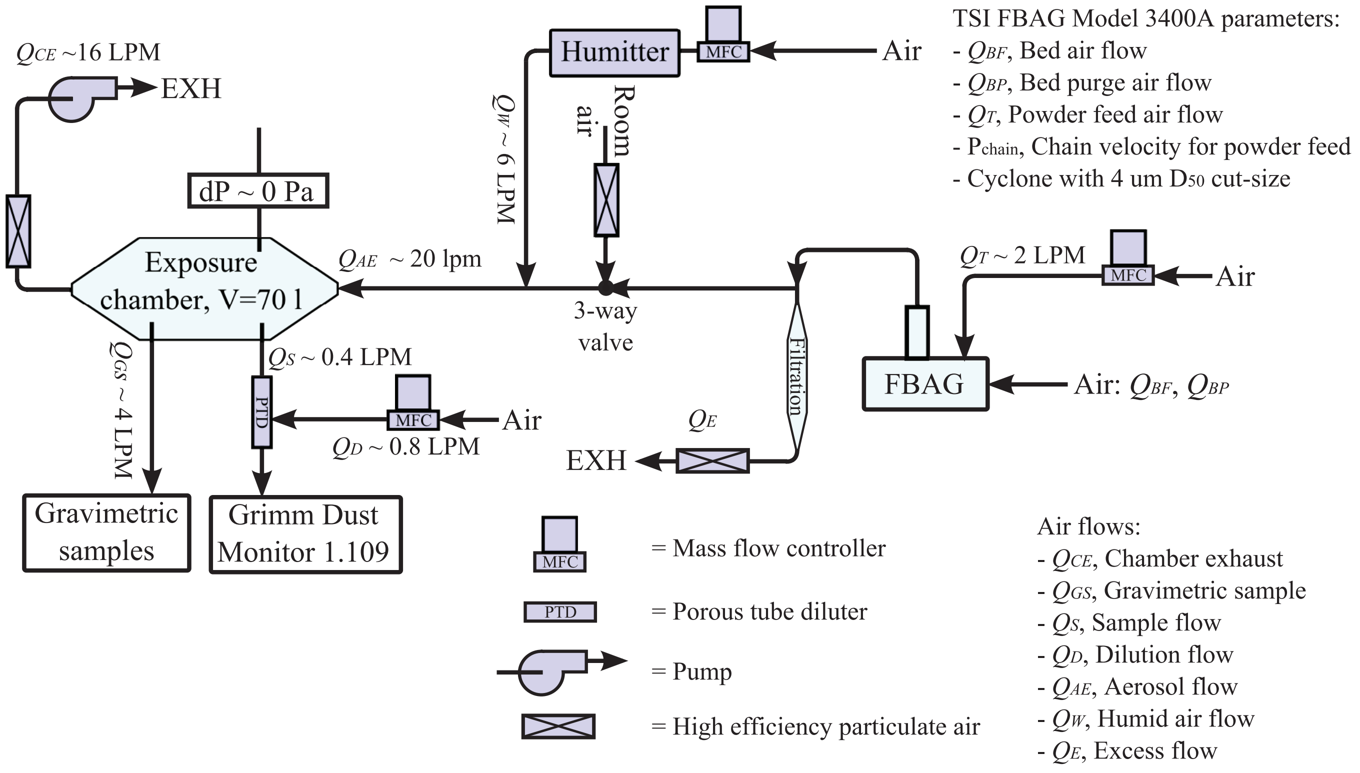

Additional file 9. Scheme of the inhalation system for exposing mice to carbon nanotubes with essential parameters and typical flow values.

In the fluidized bed aerosol generator (FBAG), CNTs are transported to the fluidizing bed with a chain where bronze pellets together with air flow (Q_{BF}) mechanically break material agglomerates. CNT feed to fluidizing bed is enhanced with additional air flow (Q_T). Thereafter, aerosolized CNT are directed to whole-body inhalation exposure camber.
